# DNA and Flavonoids Leach out from Active Nuclei of *Taxus* and *Tsuga* after Extreme Climate Stresses

**DOI:** 10.3390/plants4030710

**Published:** 2015-09-21

**Authors:** Walter Feucht, Markus Schmid, Dieter Treutter

**Affiliations:** 1 Unit Fruit Science, Center of Life and Food Sciences Weihenstephan, Technische Universität München, Dürnast 2, Freising 85354, Germany; E-Mail: Obstbau@wzw.tum.de; 2 Fraunhofer Institute for Process Engineering and Packaging IVV, Giggenhauser Street 35, Freising 85354, Germany; E-Mail: Markus.Schmid@ivv.Fraunhofer.de; 3 Chair of Food Packaging Technology, Technische Universität München, Weihenstephaner Steig 22, Freising 85354, Germany

**Keywords:** flavonoids, climate stress, *Taxus*, *Tsuga*, DNA, leaching

## Abstract

Severe over-stresses of climate caused dramatic changes in the intracellular distribution of the flavonoids. This was studied in needles from the current year’s growth of the following species and varieties: *Tsuga canadensis*, *Taxus baccata*, *T. aurea*, *T. repens*, *T. nana*, and *T. compacta*. The mode of steady changes in flavonoids was evaluated by microscopic techniques. Most of the flavonoids stain visibly yellow by themselves. The colorless flavanol subgroup can be stained blue by the DMACA reagent. In mid-summer 2013, outstanding high temperatures and intense photo-oxidative irradiation caused in a free-standing tree of *Taxus baccata* dramatic heat damage in a limited number of cells of the palisade layers. In these cells, the cytoplasm was burned brown. However, the nucleus maintained its healthy “blue” colored appearance which apparently was a result of antioxidant barrier effects by these flavanols. In late May 2014, excessive rainfall greatly affected all study trees. Collectively, in all study trees, a limited number of the mesophyll nuclei from the needless grown in 2013 and 2014 became overly turgid, enlarged in size and the flavanols leached outward through the damaged nuclear membranes. This diffusive stress event was followed one to three days later by a similar efflux of DNA. Such a complete dissolution of the nuclei in young tissues was the most spectacular phenomenon of the present study. As a common feature, leaching of both flavanols and DNA was markedly enhanced with increasing size and age of the cells. There is evidence that signalling flavonoids are sensitized to provide in nuclei and cytoplasm multiple mutual protective mechanisms. However, this well-orchestrated flavonoid system is broken down by extreme climate events.

## 1. Introduction

Histological and kinetic research with conifer species showed flavanols to be associated with nuclear histones [1,2]. By applying sophisticated physical two-photon excitation techniques the association of flavanols to nuclei could be fully confirmed [3]. A number of different flavonoids located in the cytoplasm are known to be closely linked with protection against radiation damage [4]. For example, protective quercetin glycosides were accumulated in the sun-exposed skin of distinct apple varieties [5]. The leaves of broccoli exposed to drought and water-logging responded with increased biosynthesis of kaempferol derivatives [6]. Multiple roles of flavonoids as regulators of the plant metabolism were described by Taylor and Grotewold [7]. Hereby, the structurally diverse proteins interact with variable physico-chemical properties of flavonoids to yield distinct binding types with different affinities. In this context, the question whether flavonoids are more important as antioxidants or as signalling compounds was discussed in the scientific literature. Especially (−)-epicatechin and related proanthocyanidins modulate cell signalling which is often combined with antioxidant actions [8]. If concerning the variable expression of flavonoids even inside of plant nuclei, then, signalling functions altering DNA-protein complexes should be of basic importance [9].

The many defence mechanisms of flavonoids against pathogens were discussed by Treutter [10]. A loss of vital photosynthetic processes in trees by heat and drought was summarized by Rennenberg *et al.* [11]. Global climate change is a challenge for many more experimental studies in the coming years. Overall, in contrast to the leaves of deciduous trees, the evergreen conifers have long-lived needles which are exposed over four or more years to environmental stresses. In 2013 and 2014, a number of *Taxus* genotypes and *Tsuga* were severely affected by heat, UV-radiation, droughty periods and water logging. The present paper tries to broaden our knowledge by describing dislocation of flavonoids within distinct cells of needles as a response to climate events 2013–2014.

## 2. Experimental Section

### 2.1. Study Trees, Canopy Structures, and Light Incidence

The trees of this field study grow in the Botanical Garden of the Technical University of Munich in Freising-Weihenstephan. The investigations were conducted with three trees of hemlock (*Tsuga canadensis* L.) having a pyramidal crown structure with a height up to 8 m. In addition, *Taxus baccata* L. and a group of a further four yew varieties with two shrubs each were investigated. The crowns of the *Taxus* bushes decreased from 3 m to 0.5 m in the following order: *Taxus baccata*, with the varieties *repens*, *aurea*, *compacta*, and *diamond nana*. *Var*. *repens* grew in a typical understorey ambient, had a very flat crown without any acrotony but in the lateral dimension the canopy extended to about 1.5 m in diameter. Branch ramification of var. *compacta* wa*s* very poor and the brush grew in a shady understorey ambient. By contrast, the branchiness of var. *nana* and of the semi-dwarfed var. *aurea* was extremely dense. The bushes of both species grew under full light conditions. Var. *compacta* and var. *nana*, the most dwarfed species, grew only three weeks per year, producing extremely short shoots only 3 to 5 mm in length.

### 2.2. Wide Fluctuations of Environmental Stress Conditions in 2013–2014

The long term mean of annual rainfall of the study site ranged between 700 and 800 mm. Both years, 2013–2014 showed a similar precipitation with about 780 mm each, but the intra-annual fluctuations of the rainy periods were very different. The soil is to be qualified as deep and loamy but still with adequate drainage because texture becomes coarser in deeper areas. The site slopes slightly to the south.

**Figure 1:**
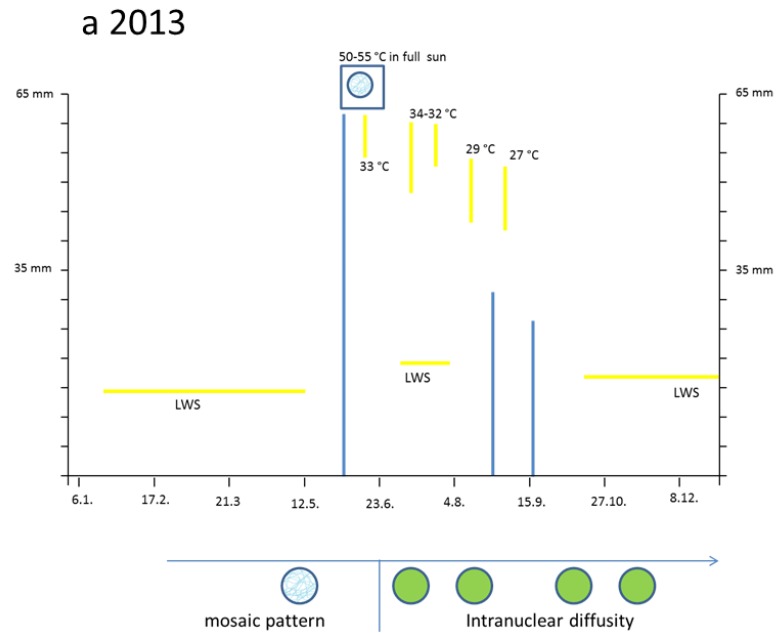

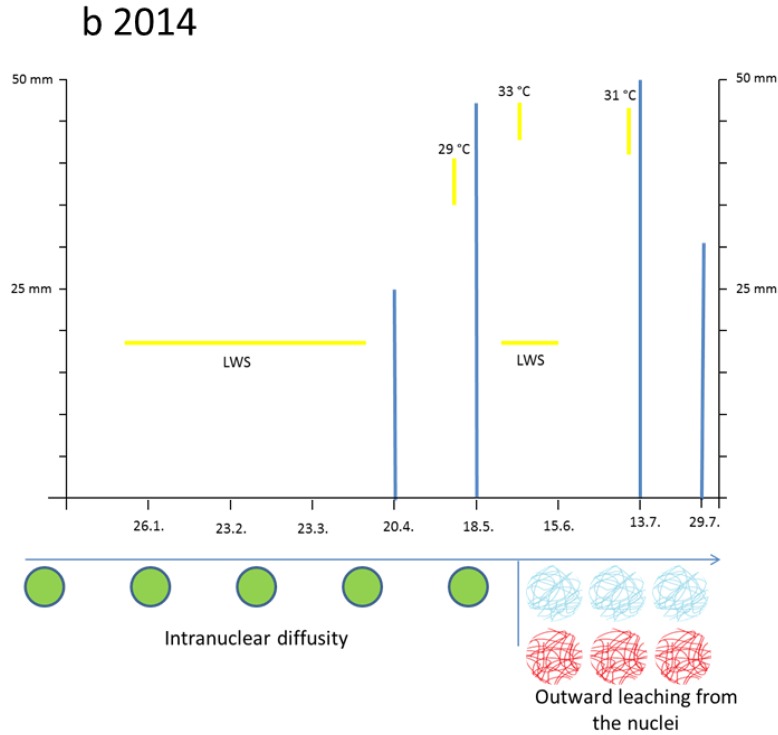
Impact of climate stress on nuclear structures of *Taxus* and *Tsuga* in the years 2013 (**a**); and 2014 (**b**). Extreme events of rainfall are shown as blue vertical lines and extreme temperatures as yellow vertical lines. Periods of very low water supply (LWS) are shown by horizontal yellow lines. When the flavanol reagent DMACA was applied, the nuclear structures are roughly characterized by blue granules or diffuse greenish colors as a mixture of blue and yellow flavonoids. The reddish colors mark nuclear DNA stained by propidium iodide.

In 2013, some periods of insufficient rainfall were major stress factors (Figure 1a). Three months before bud break (February, March, and April) were rather dry. Then, in May, only moderate rain was recorded. This period was interrupted from late May through late June by a heavy flood with about 200 mm rainfall. However, by 18 June and 27 July as well by August 3 and 5 the temperatures reached up to 32–34 °C. During late August and from mid-October to late December followed again an extended period with water deficit.

Compared with all study trees, one individual of *Taxus bacc*. was free-standing and therefore remarkably affected by heat and full sun radiation. If the thermometer was directly fixed to the sun-exposed southern side of this brush, the maximal temperatures on 20 June increased up to 50 °C from 1:00 p.m. to 5:00 p.m. Even 53 °C was measured for 30 min.

In 2014, from January to mid-April, there was a long-lasting period with rather limited water supply (Figure 1b). Then, four heavy precipitation events occurred on 21 April, on 26 May (55 mm), on 13 July (47 mm) and 29 July. A transient loss of oxygen in the soil might be suspected. The rainy periods were interrupted by three short heat periods reaching 30 °C in the shadow in 22 May, 10 June, and 19/20 July. Again, in full sun, the thermometer showed for 2 h maximal temperatures around 50 °C.

### 2.3. Tissue Sampling and Histochemical Microscopy

The investigation of the native *in planta* distribution of cellular flavonoids was performed by direct staining of fresh needle sections. Young needles from the current year growth were collected at 8 to 10 day intervals from April 2013 to November 2014. At extreme climate events, individual needles were sampled daily. For one sampling, at least five needles were used each.

After sampling, the needles were hand-cut with a razor blade in transverse sections, about 0.3 to 0.7 mm thick. Also longitudinal sections were performed to separate the epidermis from the underlying mesophyll. Thus, several thousands of cells could be studied in the two years 2013 and 2014. The most characteristic types of nuclear flavonoid expression related with climatic events were documented in microscopic pictures.

The number of nucleoli per nuclei in meristematic domains was determined by a comparative study of several years from 2002 onwards to 2014. The data were recorded by checking 250–300 cells per year.

Flavonols mostly show a more or less natural yellow staining. However, the colorless flavanols as a small subgroup of the flavonoids were stained with the specific p-dimethylaminocinnamicaldehyd (DMACA). The reagent was prepared by dissolving 1 g DMACA in 100 mL 6 N HCL + ethanol (1:1, *v*/*v*). HCl can be replaced by sulphuric acid and butanol can be used instead of ethanol. After 10–20 min, the staining reagent was withdrawn and replaced by few drops of water which caused instantly the appearance of blue stained nuclei and vacuoles. To our experience in the last 30 years, this reagent is highly specific for flavanols and their oligomeric proanthocyanidins.

However, the blue staining flavanols turned to a greenish appearance when mixed with leaching yellow flavonols. The yellow flavonoids can be intensified by diphenylboric acid 2-aminoethyl ester (Naturstoff reagent DPBA) both by light and fluorescence microscopy (365 nm). In microspore nuclei of *Taxus bacc*. both quercetin and myricetin were determined by HPLC techniques [12].

Yellow needle colors might be somewhat increased by carotenoids. It is well known that the green chloroplasts contain such carotenes. Principally, carotenes were not soluble in water. Therefore, if submerged in water few fatty mini-globules, smaller than 0.5 µm, with a nearly negligible yellow color could be detected in the greenish plastids.

Propidium iodide (Serva) dissolved in water at 10 µg/L was used to localize the nuclear DNA by intense red staining [13]. However, propidium iodide apparently yielded falsified staining intensities when DNA was faintly overlied by various phenol compounds, such as anthocyanins or kaempferol [14]. Indeed, this was likewise the case in the investigated conifer cells. Using Zeiss Axioscop and a Nicon Coolscan IV ED equipment, the nuclei stained under UV-light a bright red using 550 nm excitation with 585 nm emission filter. (The yellow flavonoids revealed a bright yellow fluorescence under UV filter G 395, FT 460, LP 470 using fluorescence light). The digital photographs were made with an Axiom Zeiss microscope and a Fujitsu-Siemens Core 2 apparatus.

Cytokinin (0.8 mM) was applied in a watery solution for 60 h to 5 cm long shoots of *Taxus baccata*, 5 cm long. The basis of three shoots was put in a reagent tube with the hormone solution. The number of blue staining nuclei of leaf sections was 30 per each shoot. Controls with a watery solution were also checked.

## 3. Results

### 3.1. Abundant Vacuolar Flavanols in the Protective Needle Tissues

**Figure 2:**
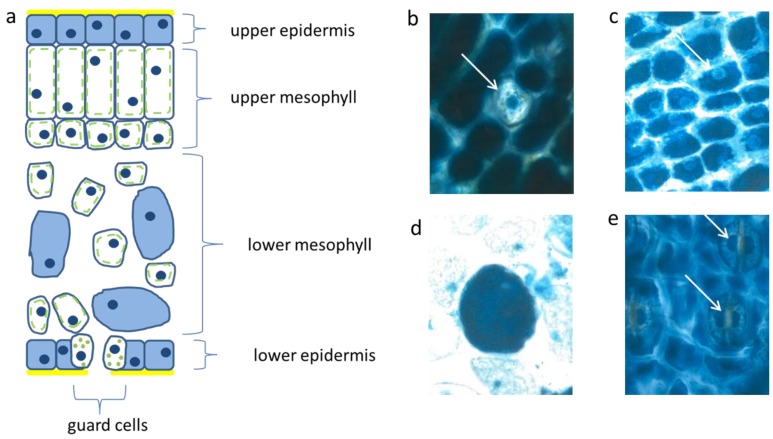
Schematic picture of a vertical section of a needle (**a**); upper epidermis (**b**,**c**); large mesophyll cell (**d**); low epidermis (**e**). Nucl. b 8 µm, c 8 µm, d 7 µm, e 9 µm elongated (white arrows).

The flat uniseriate and tangential elongated epidermis cells were rich in flavanols. This blue staining flavonoid group appeared already when the first tip of the needle arose from the sprouting bud (Figure 2a–c). Along with cell stretching, the vacuoles were filled more and more with flavanols. Thus, after about three weeks the whole surface of the needle, up to 10 mm in length, was covered with dark blue flavanols. All study trees followed this pattern, irrespective of their size and exposure to light or shady microenvironment.

The palisade layer began its growth with more or less isodiametric cells which were loaded, like the epidermis, with flavanols, but lost them along with elongation. Typically, this cell type was then fairly narrow and elongated in the vertical dimension up to 30 µm. When fully developed, the cells showed an extreme synchrony in shape and size (Figure 2a). Beneath the upper layer, there is a second palisade-like layer the cells of which show a quadratic cross section.

In the spongy parenchyma the chloroplasts developed rather early together with vacuolar flavanols. So, the beginning of photosynthesis within the needles started readily in the spongy mesophyll. This tissue was richly vacuolarized and in the rounded to ellipsoidal cells the flavanols were more prevalent than the chloroplasts (Figure 2d). In this respect, there was little variation among the different species and cultivars, except var. *aurea* and var. *compacta* (Table 1). As a rule for all species, the intercellular air-space allowing a high gaseous O_2_ diffusion between the spongy mesophyll cells was less extended in small, younger needles but became fairly large with increasing needle size and more green chloroplasts.

**Table 1:** Percent of cells filled with vacuolar flavanols as collected in mid-summer 2013 from the epidermis and the mesophyll layers. One hundred cross sections per species/varieties were investigated. (SE values of spongy mesophyll cells were calculated). Only in two species the values (b) are different from a (*p* < 0.05).

Tissues	Upper Epidermis	Palisade	Spongy	Lower Epidermis
	%	%	%	%
*Tsuga can*.	100	0	65 a	100
*Tax. bacc.*	100	0	68 a	100
*Var*. *nana*	100	0	57 a	100
*Var*. *repens*	100	0	60 a	100
*Var*. *aurea*	100	0	43 b	100
*Var*. *comp.*	100	0	81 b	100

The epidermis cells of the lower needle surface were broadly similar in shape and size to those from the upper epidermis (Figure 2e). However, the overall flavanol density of the lower epidermis was in all species somewhat less intense and more variable. This might causally be linked with the reduced incident light. Especially towards autumn and winter the flavanols were generally somewhat reduced in the lower epidermis on the whole.

Interestingly, also the guard cells contained few small vacuolar flavanol deposits about 1 µm in size and in addition even few chloroplasts. The nuclei of this cell type were generally more variable in the blue colored flavanol expression (Figure 2e).

### 3.2. Hot Spells in Summer 2013 and Adaption of the Cells

**Figure 3:**
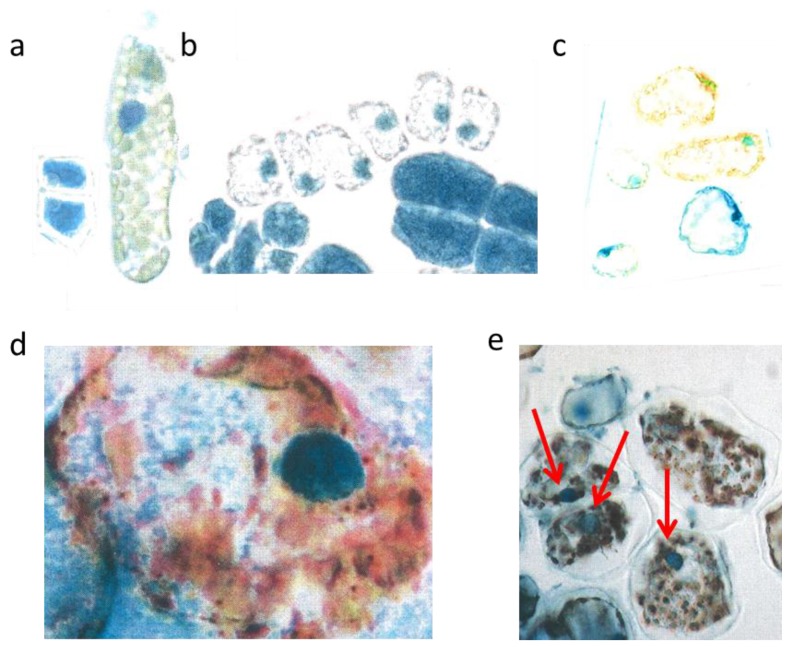
Mesophyll cells before and after burning. Two very young palisade cells were 15 µm broad (**a**, left); differentiated mesophyll cell (**b**, right) nucl. 7 µm; young mesophyll (**b** nucl. 6 µm); burned cytoplasm (**d**, nucl. 8 µm); burned chloroplasts (**e** nucl. 7 µm, red arrows).

The two small cells (Figure 3a, left) correspond to initial stages of the two palisade layers (Figure 3a, left) which later after stretching develop chloroplasts (Figure 3a, right). Why were the young palisade cells at first so blue? Each initial cell is very slender in the biophysical structures of the cell wall. Likewise, the fast elongating young epidermis cell, covering the palisade cells, carries the same implication. So, the cytoplasm of these palisade cell needed the flavanols to alleviate the dangerous UV-radiation. The change from the quadratic blue palisade cell type goes hand in hand with stretching and chloroplast formation. This process was finished after about three weeks when the needles were already 10 to 12 mm long. Then, especially the outer cell walls of the epidermis, now about 6–8 µm thick, were effective UV radiation barriers which are physically equipped with close-meshed transverse microfibrils and chemically with flavonoids.

The expanding palisade cells, as shown by a layer of six whitish lineage cells have nearly lost the flavanols, except those of the nuclei and some tiny residues along the cell walls (Figure 3b). Furthermore, some small cells of the spongy mesophyll remained already dark blue, like the four enlarged epidermal cells (Figure 3b).

However, by late May the low water supply 2013 caused first signs of a down-regulation of cellular vitality. Some cells showed an increased yellowing of the plasmalemma with remnants of blue nuclei, and other cells nearby indicated at least a blue hint of too small nuclei (Figure 3c).

The heat damage in 2013 was highest by 18 June in the free standing shrub of *Tax*. *bacc*. At the sun-exposed southern side of the canopy the temperature reached 50 °C from 1:00 p.m. to 5:00 p.m. For about 30 min up to 55 °C was measured. In the shadow, the values increased up to 30 and 32 °C.

Viewed with the naked eye, the needles remained during the following weeks with a green habit. However, by microscopy it became apparent that, in some needles of the very sun-exposed twigs distinct cells from the upper mesophyll layers, there was a brown sunburned cytoplasm (Figure 3d; the blue shreds in this Figure are from the epidermis). Probably, only distinct small sectors of the flat *Taxus* needles were exposed during mid-afternoon for a longer time to a maximal incident solar angle. However spectacularly, the nucleus located amidst the browned cytoplasm of the palisade cell stained the typical normal blue for flavanols pointing out that no damage had occurred (Figure 3d). This finding provides strong support that flavanols fulfil a crucial role as protective agents against heat stress in *Taxus* nuclei.

Also, in the upper epidermis all nuclei showed the blue protective casing. Most of these nuclei were found to be located below blue vacuole of the epidermis. So, the nuclei profit from a double security system, namely their own flavanols and those of the superimposed vacuole. The vertical extension of the epidermal vacuole overlaying the nucleus measured between 20–27 µm which is three to four times that of the nucleus with 7 µm in diameter. If during the staining procedures an epidermal vacuole accidentally was broken out, the blue nucleus within the small rim of cytoplasm became visible (Figure 2b).

Towards October 2013 roughly about 15% of the *lower mesophyll* cells located at the southern periphery of the free standing brush revealed an increasing disintegration and browning of chloroplasts (Figure 3e). Nevertheless, stable blue and compact nuclei could still be detected within the oxidized plastids.

By and large, the entire sun-exposed *Taxus* brush looked green as ever over the rest of the year. However, in the following spring, from early March to mid-April 2014, up to 20% of the shoots grown in 2013 and from the southern canopy side turned completely dark brown. If examined microscopically, all cells of the needles were completely oxidized. Obviously, a slowly progressing activity of destructive oxidative systems took place during the wintry rest period.

### 3.3. Leaching Flavanols Outwardly from the Nuclei after the Flood in Late May 2014

The first months of 2014 (January, February, and March) were droughty. During winter time the nuclei of conifers were usually hardly blue, if at all. By late April the nuclei of the newly sprouting needles attained slowly a diffuse bluish-green chromatin. Similarly, also the entire cytoplasm turned greenish as a mixture of yellow and blue. Such a curious expression of flavonoids in very young and sprouting cells was, to our 15 years of experience, quite unusual. In reality, these nuclei should reveal a blue mosaic pattern.

**Figure 4:**
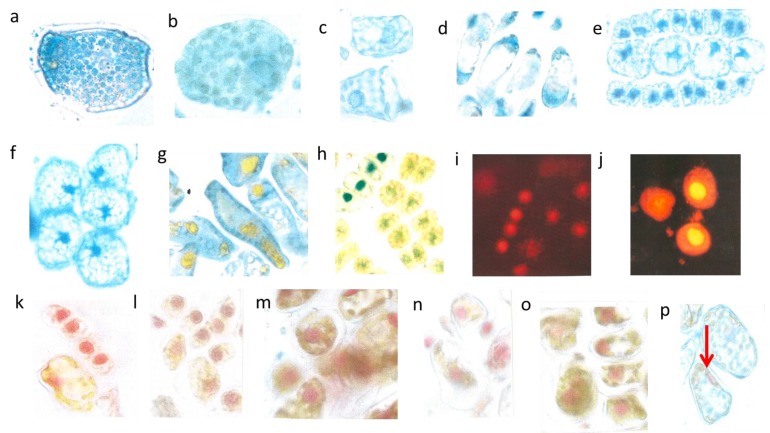
Outward leaching of flavanols and DNA from cells and nuclei. Var *repens* with flavanols covering the chloroplasts; longer diameter 42 µm (**a**); Var. *nana* with very diffused flavanol leaching of the choroplasts, longer diameter 42 µm (**b**). Mesophyll cells of *T. bacc*. (nucl.7 µm) (**c**,**d**); var. *repens* (rounded nucl 7 µm (**e**); var. *compacta* (nucl. about 7 µm (**f**); totally disappeared nuclei of var. *aurea* (**g**). Lineage cell with four compacted nuclei (nucl. 7 µm) (**h**). *T. bacc*. indicates the DNA of 7 non-leaching nuclei (7 µm) and three leaching ones 9 to 10 µm (**i**). Two nuclei have lost most of the DNA (yellow nucleus 11 µm) (**j**). Var. *aurea* with compact (7µm) non-leaching and a diffuse nucleus of a yellow mesophyll cell (**k**). Same symptoms in var. *repens*, *compact* and *nana* (**l**,**m**,**n**). The compacted nuclei measure 7 µm. The species *Tsuga can*. is equal in the patterning of leaching. Non leaching nuclei measure 7 µm (**o**,**p**).

**Figure 5:**
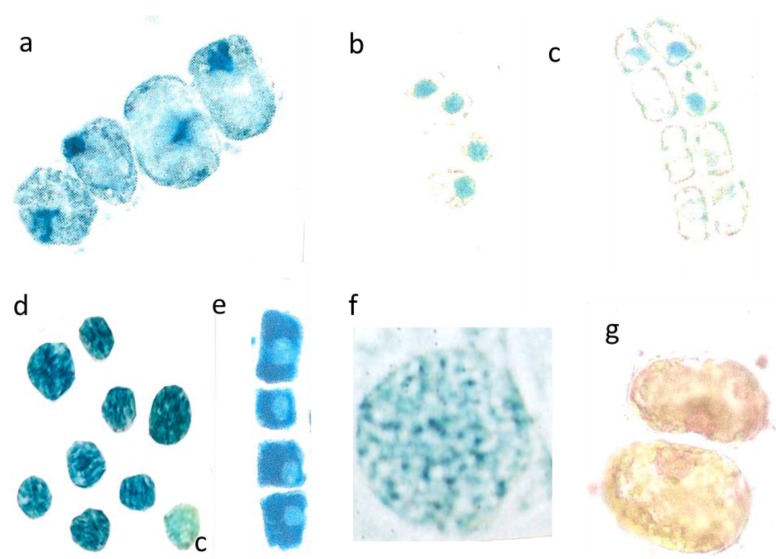
Detopping *T. bacc*. in July. Irregular size of cells and nuclei within a lineage (nucleus about 7 µm in diameter) (**a**); Nuclei with only 5 µm in diameter (**b**); Total disappearance of nuclei in five cells (nucleus 6 µm in diameter) (**c**); Application of cytokinin yields extremely dark blue nuclei (c = control is pale blue) (**d**); Dark blue lineage with pale nuclei (nucleus. 5 µm in diameter) (**e**); Normal shaped (8 µm in diameter) and activated nucleus sampled in 2012 (**f**); Pale reddish, diffuse DNA in chaotic, partially yellow cytoplasm (**g**).

Two or three days after the great flood on 26th May 2014 in a number of rather large spongy mesophyll cells the chloroplasts attained a visible blue indicative of flavanols. Again, this is an extreme and curious feature. An example of *var*. *repens* showed clear cut images of blue stained chloroplasts (Figure 4a). A further example showed *var*. *nana* (Figure 4b) with completely diffused blue staining chloroplasts. Nuclei were hardly seen. Such an unusual reaction of chloroplasts might be linked with higher flavanol synthesis during the first days of rehydration. (Stimulation of flavanol synthesis by cytokinin localized in the roots is shown in Figure 5d).

In *Tax*. *bacc*. many nuclei became water-soaked and overly turgid so that the nuclear diameter increased by about 2 µm (Table 2).Then, the flavanols began to leach out from the nuclei towards the margins of the cells. This phenomenon was clearly valid for all three trees of *Tax*. *bacc*. (Figure 4c,d). It is important to note that all study trees showed leaching of flavanols in many cells all over the entire canopies. Significant differences between light-exposed and shaded twigs or dwarf and vigorous trees were not evident. In var. *repens* (Figure 4e) the nuclear flavanols leached outwardly between the starch grains of the cytoplasm. The shapes of the nuclei were deformed in the four large lineage cells. As a rule for all study trees, the more advanced the development of a cell the more pronounced was leaching. The leaching effect as shown in var. *compacta* produced star-shaped nuclei by flux of flavanols between the adjacent starch grains (Figure 4f), but most of the flavanols were displaced towards the cell walls. Some cells of var. *aurea* showed an overall spreading of flavanols whereas the yellow phenols were strictly confined to vacuoles (Figure 4g). Var. *aurea* had a pronounced tendency to synthesize the yellow flavonoid molecules. Overall, the epidermal cell walls and to some extent the cytoplasm of the terminal needle sectors displayed a fairly yellow color (Figure 4h). As ever, the pale green, rudimentary nuclei indicate a mixture of yellow and blue. By contrast, the recently formed lineage with the four young cells still fastened to each other was equipped with fairly compact, rounded, and prominent dark blue nuclei. As already mentioned above, such a compactness of recently formed young nuclei is readily unusual for a young lineage. Really, the nuclei should be in a fine-granular mosaic state. Obviously, the presence of too intense yellow flavonoids in the cytoplasm is not compatible with blue nuclei.

In foregoing years (2001–2012), the size of conifer nuclei, if grown under normal climate conditions, proved to be very constant (Table 2). However, from day 1 to day 7 after the flood 2014, many nuclei of all species suffered from increased water uptake resulting in a larger diameter up to 10 or 12 µm. (Table 2). In *Tax*. *bacc*., about 25% of the nuclei from the summer sprouts (S-flush 2014) emerging in late July reached only 5 µm in diameter (not shown in Table 2).

**Table 2:** Range of nuclear size (µm in diameter) from the needles sampled under normal growth conditions during previous years 2001–2012. Further sampling was done after the flood in July 2014. Different letters indicate significant differences between normal and flood; (*t*-test, *p* ≤ 0.05). Number of nuclei larger than 8 µm in diameter after the flood are given in %.

	*T*. *bacc.*	Var. *aurea*	Var. *nana*	Var. *rep.*	Var. *comp*.	*Tsuga*
Normal	7–8 a	7–8 a	7–8 a	7–8 a	6–7 a	7–8 a
Flood	7–12 b	7–10 a	7–9 a	7–9 a	6–9 a	7–12 a
>8 µm (%)	42	12	17	21	9	15

### 3.4. Outward Leaching of DNA from the Nuclei after the Flood in Late May 2014

In all trees of the present study, the nuclear DNA from many mesophyll cells diffused away. The needles were grown in 2013 and 2014. A nuclear size surpassing 8 µm in diameter was a sensitive index of higher leaching susceptibility. Concrete data were shown for *Tax*. *bacc*. and *Tsuga* (Table 3).

**Table 3:** Percent of cells with still well developed, non-leaching nuclei after the flooding stress as recorded in August-September 2014. Each average value for flavanols and DNA is the mean of 40 observed needles (the four cultivars of *Taxus* varied approximately similar to *Tax*. *bacc*). The difference between epidermal and mesophyll layers is indicated by different letters within the same column (*t* test, *p* ≤ 0.05).

Compound	Flavanol		DNA	
	*Tax. bacc*.	*Tsuga*	*Tax. bacc*.	*Tsuga*
Upper epidermis	100 a	100 a	100 a	100 a
Upper mesophyll	85 b	86 b	84 b	82 b
Lower mesophyll	83 b	88 b	83 b	85 b
Lower epidermis	100 a	100 a	100 a	100 a

To begin with *Tax*. *bacc*., only one or two days after the loss of flavanols also DNA was found to leach out from the nuclei. Young four-celled lineages showed still intact nuclei and revealed a bright red fluorescence (Figure 4i). The diameter of the four intact nuclei was 7 µm each. However, three somewhat older single cells showed diffuse pale red nuclei about 10 µm in diameter (Figure 4i). Also in *Tax*. *bacc*., the outward leaching of DNA from the nuclei into the cytoplasm was more precisely demonstrated by the red and yellowish flavonoid fluorescence (Figure 4j).The two more yellowish nuclei had already lost most of the DNA.

In var. *aurea* (Figure 4k), the four obviously intact lineage nuclei stained a correct and clear-cut rosy red for DNAs, but the adjacent single and enlarged cell with a yellowish cytoplasm yielded a diffuse pale reddish nucleus. In the following example from var. *repens*, the red tint of nuclear DNA appeared to be somewhat intermixed with a pale yellow and three nuclei were already diffused (Figure 4l). In the needles of var. *comp*. (Figure 4m) cell clusters were found with a mixture of reddish and brownish colors. Finally, var. *nana* showed large differences from cell to cell regarding diffusing DNA (Figure 4n).

Leaching symptoms of DNA in enlarged cells with a yellow cytoplasm were also observed in *Tsuga*, in contrast to the four young intact lineage cells located nearby (Figure 4o). Sometimes the chloroplasts of *Tsuga* turned to blue colors of flavanols (Figure 4p). Suggestively, they were leached out from the nuclei. In one of the two cells a rather pale reddish DNA was pressed towards the cell wall. In the second cell there was no more any reddish tint of DNA.

To sum up, in all study trees about 15% of the mesophyll cells were affected by leaching in contrast to the upper and lower epidermis. This is exemplarily shown for *Tax*. *bacc*. and *Tsuga* (Table 3).

### 3.5. Breakdown of Cell Cycling at the Start of the Summer Flush 2014

Detopping of *Tax*. *bacc*. in July is a common practice to stimulate the branchiness and density of the bushes growing in a garden. Then, the newly emerging summer flushes normally reached about 5 to 10 cm in length. However, detopping in July 2014 resulted in disturbed mitosis even during the first cell divisions. The young coppices started off slowly and the mitotic cells showed a series of structural failures. Finally, the shoots tapered off stunting at 4–6 mm in length. The cells, if too small and lacking DNA synthesis in S-phase did not go on to divide after about five days.

The four newly formed lineage cells from *Tax*. *bacc*. (Figure 5a) were not correctly synchronized in size and shape. An imbalance of mutual signalling is evident. Normally, conifer nuclei are strictly spheroid. However, the mal-shaped and diffuse nuclei, instead of being located in the central cell position were attached to the cell walls as a sign of silencing. Further division is then not possible. The many diffuse blue patchy flavanols all over the four cells should be enclosed in well-defined vacuoles. There is a severe lack of internal cell organization.

In a further example, the nuclei of four very small lineage cells were still spheroid, but only 5 µm in diameter instead of 7 µm and embedded in a diffuse yellow cytoplasm (Figure 5b). In two closely parallel located lineages (Figure 5c) with four cells each, five or six of the eight nuclei failed to produce the obligatory blue staining flavanols. In one case there is only a residual blue of the nucleus. Such a drastic stop in developing nuclear flavanols of a cell lineage was never observed in our previous long-term investigations since 2000.

When cytokinin (0.8 mM in water) was added to such needles with very poorly staining nuclei, then the nuclear flavanols were densely colored. The non-treated control nucleus (co) is only pale blue **(**Figure 5d). In a further four-celled lineage, the addition of cytokinin resulted overall in a dark blue cytoplasm but in rather pale blue nuclei as a very curious response (Figure 5e). To give an example of a perfect nucleus with its granulated structures of euchromatin and heterochromatin, it is necessary to pick up an active nucleus of previous investigations in 2012 (Figure 5f). Also nuclear DNA molecules of the summer sprouts showed all over the cell as a diffuse red staining mixed with yellow leaching flavonoids (Figure 5g).

**Figure 6:**
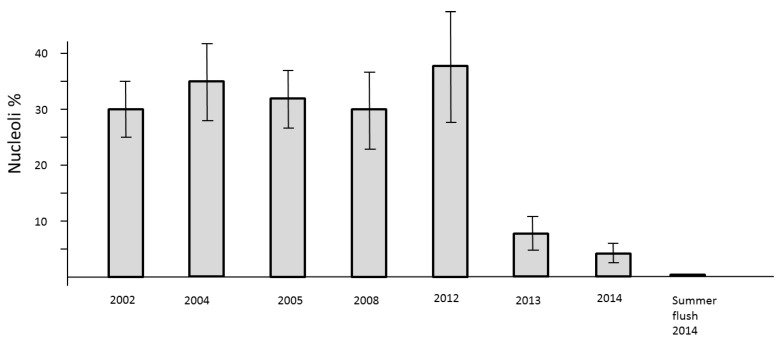
Number of active nucleoli per nuclei in mitotic cell clusters of needles in previous years 2002, 2004, 2005, 2008, and 2012 as compared with 2013–2014.

The loss of mitotic activity is well known to be linked with a down-regulation of transcription. In the nuclei such a process is structurally clearly evidenced by the drastic reduction of clearly visible nucleoli. Usually, the nuclei of all study trees are about 2 µm in diameter. They may be less than 1 µm in size and therefore difficult to detect. Consequently, a low level of nucleolar activity und thus decline of RNA synthesis slows down the processes of cell cycling but the formation of new cells is not stopped. This phenomenon was observed in springtime 2013 and 2014. A definite stop of cell division was recognized during the summer-flush 2014 (Figure 6).

## 4. Discussion

### 4.1. Strategic Distribution of Protective, Antioxidant Flavanols in Needles

Excess of UV light and heat causes an inhibition of electron transport as well as reduced photosynthesis and inactivation of many chloroplast proteins [15,16]. According to Fischbach *et al.* [17], UV-radiation reduced elongation and biomass of needles from Norway spruce. As a response mechanism, the flavonoids of the upper epidermis of plants can attenuate UV-B radiation by about 75% to 95% [18]. Importantly, the histological studies revealed a very critical phase of the needles during the intense growth period in May as the dividing and stretching epidermis cells have not yet established enough structural thickness to achieve full protective capacity for the downward adjoining cells.

Principally, the strategic optimal position of preformed defence phenols in distinct cell layers, like the upper epidermis, is a fundamental evolutionary adaption to radiation stress [19]. In all trees of the present investigation it could be shown that vacuoles being fully packed with flavanols occupied the entire epidermis cells. The blue nuclei of the upper epidermis are hidden mostly underneath the large vacuoles. The fully developed epidermal barrier apparently protects the underneath located vertically stretched photosynthetic palisade cells which are devoid of vacuolar defence flavanols. So, there is more space for a higher number of chloroplasts and intensification of photosynthesis. Only the nucleus as the regulatory key centre of each cell reasonably retains its own flavanol-based blue barrier in the palisade layers. The naked flavanol-free nucleus apparently is no insurance for survival.

After the heat shock 2013, in few of these upper palisade cells the cytoplasm was burned brown. At 50 °C, there was an oxidative breakdown of the cytoplasm antioxidant system, except the nucleus. The intensely blue colored nuclear anti-stress flavanols obviously prevented oxidative heat burning. Overall, DNA is rapidly damaged by UV radiation [20].

It is well-known that flavanols in watery solutions oxidize rapidly. However, oxidative browning reactions of flavanols were impeded if they were bound to nuclear histones [1]. Also DNA is very sensitive to oxidation [21]. Thus, a tight oxygen-free attachment between flavanols, histones and DNA is readily a key essential feature. In this context, other flavonoids, such as the antioxidants kaempferol or rutin, were found to be also attached to the histones of microspores from conifers [12].

The burned cytoplasm and chloroplasts of palisade cells as found in very heat-exposed needles of *Taxus bacc*. point to a definite limit in a defence capacity against extreme radiation stress. This species displays a decrease in the efficiency of photosystem II when growing in a high light environment [22].

Regarding the spongy mesophyll, there are very conspicuous large cells completely filled with dark blue vacuolar flavanols. However, the number and size are very variable in all study trees. In physiological terms, this means that also the mesophyll needs many flavanols to prevent oxidation especially of the nuclei. Following Fini *et al.* [23], reactive oxygen species move from chloroplasts into adjacent vacuoles where flavonoids are accumulated to scavenge the toxic radicals. If this is so in *Taxus* and *Tsuga*, then the costly synthesis of large flavanol cells in the lower needle mesophyll is understandable.

The lower epidermis of all study trees was not always as blue as the upper one. This may be because less sunlight stress at this needle site allowed some reduction of the costly flavanol synthesis.

### 4.2. Extreme Climate Events and the Epigenetic Response of the Nuclei

Principally, heat shocks can block distinct transcription factors [24]. Overall, proteins can be degraded by extreme heat [25]. During the less severe drought events in 2003, 2007 and 2010 the nuclear flavanols disappeared only for some days without any visible leaching and thereafter returned to their normal blue habit [9].

By contrast, the nuclear flavanols did not disappear 2013 during the extreme heat shock on 18 June within the burned cytoplasm. Apparently, epigenetic cell signalling to the nuclei which induce gene expression for flavanols synthesis gave so much alarm in June 2013 that the nuclei were perfectly shielded. Obviously in the case of *Taxus*, the genes for blue flavanol-colored nuclei can be activated or blocked depending on the climate conditions [9]. In this context, the nuclear flavanols are most likely synthesized at those parts of the endoplasmic reticulum (ER) which is aggregated directly to the outer nuclear membrane.

The first four months of 2014, from January to late April, were likewise fairly droughty. Principally, a certain decline in cellular hydration might result in an increase of toxic oxygen radicals [26], and it is generally accepted that drought elevates the levels of abscisic acid which in turn promotes ageing. Destructive oxidative ageing after treatment with paraquat was impeded in tissues of sweet cherry by addition of flavanols [27]. In view of the drought-stressed needles with notable flavanols being attached even to the chloroplasts of *Tax. bacc.* (Figure 4a,p), self-regulated attraction flavonoid as antioxidants is evident [16,23].

Normally, during less severe and short drought periods as for example in May and June 2008 the nuclear flavanols disappeared only for some days but without any leaching and then returned to the nuclei [9].

However, few days after the flood in late May 2014 a dramatic efflux of nuclear flavanols and DNA was observed. This reaction is comparable to the situation in pea roots described by Gladish *et al.* [28] and Niki and Gladish [29] in that extreme flooding combined with high temperatures produced apoptotic-like symptoms and induced fragmentation of nuclear DNA. Obviously, in the conifers as well as in pea, the structural integrity of cytoplasm and nucleus was strongly weakened. Degenerative membrane structures are observed after photo-oxidative stress when chloroplasts are injured [30]. Apart from the flavanols, also quercetin derivatives protect the cells against both UV radiation and oxidative damage as shown in leaves of *Petunia* [4]. Returning to the flooded conifer cells, it is of particular importance that under flooding, zeatin is easily oxidized in *Zea mais* by cytokinin oxidase [31]. It has been long known that damage of membranes, as induced by a shortage of cytokinins, leads to a loss of membrane functions [32].

Droughty and extreme rainy periods as was typical for 2013–2014, greatly affected the organelles of the cytoplasm. Consequently, the yellowed chloroplasts have reduced their activity. Chlorosis is an indicator of senescence and readily the cytokinins could recover a certain activity of the affected tissues. Virtually this growth hormone is capable of recovering faded levels of the beneficial nuclear flavan-3-ols to densely blue colors (Figure 5d). It appears that the intimate cooperation of flavanols and cytokinin [9] is a fundamental aspect to an understanding of the growth potential and defence conditions of the study trees.

### 4.3. Final Senescence in Mid-Summer 2014

After the heat spells in mid-summer 2014, the brushes of *Tax bacc.* suffered in late July from an extremely poor resprouting and mitotic disaster of coppice shoots. All other study trees showed no resprouting at all. This final breakdown of activity is, as shown in the foregoing chapters, the result of a continuous weakening of the photosynthetic equipment. The chloroplasts and mitochondria are influenced by intricate, retrograde signaling with the nuclei [33]. Over-optimum temperatures resulted in a poor transcription of ribosomal genes and a decline of protein synthesis [34]. This fact points to the intimate link between nucleolar activity and transcription of RNAs. The increasing frequency of nucleoli per nuclei is a true indicator for transcriptional access to DNA sequences and ribosomal protein synthesis in mitotic cell systems [35]. At this point it should be emphasized that the use of the blue staining flavanol reagent is an efficient screen for nucleoli because they remain colorless within the blue nucleoplasm. Diffuse, evenly stained, and compacted inactive nuclei indicate a too strong transcriptional repression [36]. Since 2002 down-regulation of the nuclear activity was not as evident as in 2013–2014.

All in all, the degree of extreme climate stress events can be checked by the intracellular mismatch distribution of the flavonoids. Hereby, the conifers try to activate flavonoids as signalling modulators to alleviate the multiple types of oxidative stress [37]. Following Morgan [38], specified molecules concentrate under evolutionary aspects in distinct tissues or subcellular plastids where they can operate most effectively. The role of distinct flavonoids in conifer needles growing under proceeding climate stress conditions certainly confirms this view.
